# Incidence of Post-Traumatic Stress Disorder After Road Traffic Accident

**DOI:** 10.3389/fpsyt.2019.00519

**Published:** 2019-07-19

**Authors:** Wubalem Fekadu, Tesfa Mekonen, Habte Belete, Amsalu Belete, Kalkidan Yohannes

**Affiliations:** ^1^Psychiatry Department, School of Medicine, College of Medicine and Health Sciences, Bahir Dar University, Bahir Dar, Ethiopia; ^2^Psychiatry Department, School of Medicine, College of Health Sciences, Addis Ababa University, Addis Ababa, Ethiopia; ^3^College of Health Sciences, Debre Tabor University, Debre Tabor, Ethiopia; ^4^Department of Psychiatry, College of Medicine and Health Sciences, Dilla University, Dilla, Dilla, Ethiopia

**Keywords:** PTSD (post traumatic stress disorder), incidence, car accident, predictors, Ethiopia

## Abstract

**Background:** Post-traumatic stress disorder (PTSD) occurs after exposure to actual or threatened death, serious injury, or sexual violence. Road traffic accident (RTA) is one of the traumatic experiences, which may result in PTSD. But treatment is mainly concentrated on physical health. This may be due to a lack of evidence in low-income countries.

**Aim:** To determine the incidence level and identify risk factors of PTSD after RTA.

**Methods:** Longitudinal panel study was done to assess the incidence of PTSD after RTA. The study was conducted in three orthopedic settings of Bahir Dar town Northwest, Ethiopia. The study was on 299 adult car accident survivors. PTSD Checklist (PCL) civilian version, Sheehan disability assessment scale, Patient Health Question (PHQ-2), and Alcohol Use Disorder Identification Test (AUDIT) were instruments to assess the outcome and associated factors. The generalized linear model with Poisson log-linear method was applied to identify associated factors. Ethical clearance was obtained from Bahir Dar University. Individuals with PTSD symptoms were linked to the psychiatric clinic.

**Result:** One hundred thirty-nine (46.5%) participants had at least three extremely severe symptoms that fulfil criteria B, C, and D of Diagnostic Statistical Manual IV of PTSD. The most frequent severe symptoms were having repeated, disturbing memories, thoughts, or images. Two hundred ten (70.2%) participants reported the extreme impact of the accident on work or schooling and 156 (51.9%) reported extreme problems in social functioning. Alcohol dependence, hazardous alcohol consumption, and harmful use were reported by 7.9%, 15.1%, and 4.7% of the participants, respectively. In the final model witnessing death, severe sleep problem and severe impairment in family functioning were significantly associated with PTSD.

**Conclusion:** Nearly half of RTA survivors develop PTSD. Clinicians need to link these patients to the psychiatry clinic. Special attention should be given to patients who witnessed death, with a serious disability, and previous psychiatric history.

## Introduction

Post-traumatic stress disorder (PTSD) occurs after exposure to actual or threatened death, serious injury, or sexual violence. It is characterized by recurrent, involuntary, and intrusive distressing memories of the traumatic event and dissociative reactions (1). PTSD symptoms are higher in traumatic events compared to non-traumatic events (2).

Road traffic accident (RTA) is one of the traumatic experiences, which may results in PTSD (3, 4). Globally, road traffic injuries result in more than 1.25 million deaths and 50 million injuries per year (5). More than 90% of road traffic deaths occur in low- and middle-income countries (5). In Ethiopia, though the number of vehicles is one of the lowest in the world, morbidity, disability, and mortality are high. In the region, where the current study is conducted, at least one person is killed each day and 1,884 people are injured or disabled per year due to RTA (6).

The incidence, prevalence, and severity of PTSD in peoples who witnessed or survived from RTA depend on sex, age, place of injury, perceived life treat, and responsibility for the injury (7, 8). Santiago et al. (9) have reported a prevalence of 30.1% at month 1 and 14.0% at month 12 in non-intentional injuries from a systematic review of 58 articles. A study in Sweden reported prevalence of 19.7% in women and 13.2% in men PTSD (10). In a follow-up study, 18.4% of survivors fulfill the criteria for PTSD within 6 months after the accident in Germany (11). In Nigeria, PTSD was higher in RTA survivors (26.7%) than two matched controls (8% and 8.7%) (12). Pervanidou et al. (13) in Greece have reported a higher (nearly half) incidence of PTSD in children who survived from motor vehicle accident.

The important predictor about the level of PTSD across studies was severity of injury. Most articles reported higher magnitude of PTSD in severe cases while some report insignificant association. Keppel et al. (14) and Mayou et al. (15) have reported higher level of PTSD in severe injuries while Stallard et al. (16) reported no association between the two.

Though PTSD was common in victims of RTA, the treatment options are mainly focused in restoring physical activity. The stress is either left undiagnosed or left untreated. If PTSD is left untreated, it will reduce quality of life, limit physical rehabilitation, and increase hospital stay (11, 17). This may be due to lack of evidences especially from low-income countries. The aim of this study was to determine the incidence level and identify factors associated with PTSD in individuals who survived from RTA.

## Methods

The study is reported based on STROBE (Strengthening the Reporting of Observational Studies in Epidemiology) statement, which guides reporting of Observational Studies (18). This is an incidence report from individuals surviving RTA. The study was conducted in three orthopedic settings of Bahir Dar town Northwest, Ethiopia. One of the hospitals (Felegehiwote referral hospital) is a public regional referral hospital while the other two (Gambi and Addinas hospitals) are private hospitals.

### Participants

Adults who survived from RTA within 1 month who

1. Have no major trauma before the RTA
2. Can communicate and with no major cognitive impairment which limits verbal report of the symptoms
3. Are admitted to one of the hospitals for orthopedic or other medical care. This is done because we believe that the accident may be enough to result in PTSD if the patient is admitted to a hospital.

Three hundred thirteen adult RTA survivors who had been admitted in one of orthopedic setting of Bahir Dar town were approached for interview, and 299 (95.53%) accepted to participate. One hundred ninety-two (64.2%) were males, and the median age was 31 with Inter Quartile Range (IQR) of 25–42. One hundred thirty-five (45.2%) of the participants were married, and 28.1% are illiterate. The median household income was 1,000 birr with average family members of 3.46 (**Table 1**). Two hundred sixteen (72.2%) of the survivors were travelers during the accident, and the remaining were pedestrians. One hundred twenty-eight (42.8%) of the participants witnessed a death in the accident, and 22.4% had other family member in the accident.

**Table 1:** Demographic characteristics of RTA survivors in orthopedic settings of Bahir Dar, Ethiopia.

Variable	Responses	Frequency (%)
Sex	Male	192 (64.2)
	Female	107 (35.8)
Religion	Orthodox	249 (83.3)
	Muslim	34 (11.4)
	Protestant	14 (4.7)
	Catholic	2(0.7)
Marital status	Single	108 (36.1)
	Divorced	20 (6.7)
	Widowed	22 (7.4)
	Separated	14 (4.7)
	Married	135 (45.2)
Residence	Rural	108 (36.1)
	Urban	191 (63.9)
Occupation	Gov’t employ	46 (15.4)
	Private employ	56 (18.7)
	Merchant	46 (15.4)
	Farmer	60 (20.1)
	Housewife	30 (10.0)
	Daily worker	10 (3.3)
	Student	46 (15.4)
	Unemployed	5 (1.7)

#### Instruments

1. PTSD Checklist (PCL) civilian version, a self-report scale with 17 items having five-point severity scale, was used to assess symptoms of PTSD. It had good psychometric properties including test–retest reliability value of 96% (19). The tool had been developed based on the Diagnostic Statistical Manual symptoms of PTSD (1). The tool was not validated in Ethiopia, but we have done pre-test and confirmatory factor analysis (21) to see the performance of the tool. In the confirmatory factor analysis, the first two items were highly correlated (82.2%) and one of the items was deleted to avoid multicollinearity. The final 16-item instrument had Cronbach’s alpha (22) of 96.2% (**Supplementary File**). PTSD was reported if an individual report:
  - At least one response of extremely severe symptom in questions 1–5 and
  - At least one response of extremely severe symptom in questions 6–12 and
  - At least one response of extremely severe symptom in questions 13–17
2. Sheehan disability assessment scale (23) was used to screen disability related with the trauma.
3. Patient Health Questionnaire (PHQ-2) was used to assess core symptoms of depression. PHQ-2 was validated in Ethiopia to screen depression (24). The other instruments were Oslo social support scale (25), Alcohol Use Disorders Identification Test (AUDIT) (26), and 8-item PROMIS Sleep Disturbance Short Form (27).

Data were collected with trained nurses, and mental health professionals closely supervised the data collection. The interview was done with Amharic (local language). Data entry was done with Epi Info-7 (28), and SPSS (29) was used to analyze the data. Generalized linear model with Poisson log-linear method (30) was applied to identify factors associated. The presence of association was presented with risk ratio (31) and 95% confidence interval where p-value ≤ 0.05 is considered as statistically significant.

## Results

### Incidence of PTSD

One hundred thirty-nine (46.5%) participants had at least three extremely severe symptoms of PTSD, which fulfills criteria B, C, nd D of DSM IVRT criteria of PTSD (1). The most frequent severe symptoms were having repeated, disturbing memories, thoughts, or images of a stressful experience from the past and feeling jumpy or easily startled, while feeling emotionally numb or being unable to have loving feelings for closed ones and avoiding thinking about or talking about a stressful experience from the past or avoid having feelings related to it are rare severe symptoms (**Table 2**).

**Table 2:** PTSD item responses among RTA survivors in orthopedic settings of Bahir Dar, Ethiopia.

Questions	Not at all	A little bit	Moderately	Quite a bit	Extremely
Repeated, disturbing memories, thoughts, or images of a stressful experience from the past?	42 (14.0)	20 (6.7)	36 (12.0)	90 (30.1)	111 (37.1)
Suddenly acting or feeling as if a stressful Experiences were happening again (as if you were reliving it)?	68 (22.7)	13 (4.3)	43 (14.4)	106 (35.5)	69 (23.1)
Feeling very upset when something reminded you of a stressful experience from the past?	55 (18.4)	26 (8.7)	51 (17.1)	96 (32.1)	71 (23.7)
Having physical reactions (e.g., heart pounding, trouble breathing, or sweating) when something reminded you of a stressful experience from the past?	83 (27.8)	18 (6.0)	49 (16.4)	84 (28.1)	65 (21.7)
Avoid thinking about or talking about a stressful experience from the past or avoid having feelings related to it?	60 (20.1)	35 (11.7)	66 (22.1)	78 (26.1)	60 (20.1)
Avoiding activities or situations because the reminded you of a stressful experience?	63 (21.1)	23 (8.1)	69 (23.1)	73 (24.4)	71 (23.7)
Trouble remembering important parts of a stressful experience?	76 (25.4)	22 (7.4)	53 (17.7)	79 (26.4)	69 (23.1)
Loss of interest in activities that you used to enjoy?	48 (16.1)	29 (9.7)	67 (22.4)	93 (31.1)	62 (20.7)
Feeling distant or cut off from other people?	69 (23.1)	22 (7.4)	59 (19.7)	77 (25.8)	72 (24.1)
Feeling emotionally numb or being unable to have loving feelings for those close to you?	58 (19.4)	32 (10.7)	55 (18.4)	93 (31.1)	61 (20.4)
Feeling as if your future will somehow be cut short?	49 (16.4)	25 (8.4)	82 (27.4)	76 (25.4)	67 (22.4)
Trouble falling or staying asleep?	37 (12.4)	30 (10.0)	70 (23.4)	91 (30.4)	71 (23.7)
Feeling irritable or having angry outbursts?	44 (14.7)	29 (9.7)	77 (25.8)	75 (25.1)	74 (24.7)
Having difficulty concentrating?	83 (27.8)	21 (7.0)	51 (17.1)	68 (22.7)	76 (25.4)
Being “super-alert” or watchful or on guard?	33 (11.0)	21 (7.0)	65 (21.7)	92 (30.8)	88 (29.4)
Feeling jumpy or easily startled?	86 (28.8)	9 (3.0)	46 (15.4)	50 (16.7)	108 (36.1)

### Impact of the Trauma (Sheehan Disability Assessment Scale)

Two hundred ten (70.2%) participants report extreme impact of accident on work or schooling, and 156 (51.9%) report extreme problems in social functioning (**Table 3**).

**Table 3:** Disability assessment among RTA survivors in orthopedic settings of Bahir Dar, Ethiopia.

Impact level		Work/school	Social life	Family life/home responsibilities
Mild	1	3 (1.0)	2 (0.7)	25 (8.4)
	2	1 (0.3)	2 (0.7)	11 (3.7)
	3	2 (0.7)	3 (1.0)	16 (5.4)
Moderate	4	6 (2.0)	11 (3.7)	9 (3.0)
	5	3 (1.0)	11 (3.7)	11 (3.7)
	6	8 (2.7)	26 (8.7)	19 (6.4)
Markedly	7	15 (5.0)	24 (8.0)	17 (5.7)
	8	14 (4.7)	27 (9.0)	15 (5.0)
	9	37 (12.4)	37 (12.4)	30 (10.0)
Extreme	10	210 (70.2)	156 (51.9)	146 (48.8)

### Alcohol and Sleep-Related Problems

Alcohol dependence, hazardous alcohol consumption, and harmful use were reported by 7.9%, 15.1%, and 4.7% of the participants, respectively (**Table 4**). Thirty-three (11%) participants reported moderate sleep disturbance. One hundred twenty-four (41.5%) accident survivors report mild sleep disturbance, and the remaining 47.5% had none to slight sleep disturbance (**Table 5**).

**Table 4:** Alcohol-related problems among RTA survivors in orthopedic settings of Bahir Dar, Ethiopia.

S. No	Questions	Responses				
		**Never**	**Monthly or less**	**2–4 times a month**	**2–3 times a week**	**Four or more times a week**
1	How often do you have a drink containing alcohol?	156 (52.2)	13 (4.3)	10 (3.3)	91 (30.4)	29 (9.7)
		1 or 2	3 or 4	5 or 6	7 to 9	10 or more
2	How many standard drinks containing alcohol do you have on a typical day when drinking?	59 (19.7)	63 (21.1)	21 (7.0)	2(.7)	–
		**Never**	**Less than monthly**	**Monthly**	**Weekly**	**Daily or almost daily**
3	How often do you have six or more drinks on one occasion?	79 (26.4)	40 (13.4)		12 (4.0)	14 (4.7)
4	During the past year, how often have you found that you were not able to stop drinking once you had started?	65 (21.7)	45 (15.1)	13 (4.3)	13 (4.3)	7 (2.3)
5	During the past year, how often have you failed to do what was normally expected of you because of drinking?	70 (23.4)	40 (13.4)	12 (4.0)	12 (4.0)	9 (3.0)
6	During the past year, how often have you needed a drink in the morning to get yourself going after a heavy drinking session?	75 (25.1)	32 (10.7)	13 (4.3)	12 (4.0)	11 (3.7)
7	During the past year, how often have you had a feeling of guilt or remorse after drinking?	85 (28.4)	27 (9.0)	13 (4.3)	10 (3.3)	8 (2.7)
8	During the past year, have you been unable to remember what happened the night before because you had been drinking?	90 (30.1)	23 (7.7)	12 (4.0)	12 (4.0)	6 (2.0)
9	Have you or someone else been injured as QWA result of your drinking?	262 (87.6)		25 (8.4)		12 (4.0)
10	Has a relative or friend, doctor, or other health worker been concerned about your drinking or suggested you cut down?	255 (85.3)			30 (10.0)	13 (4.3)

**Table 5:** Sleep problems among RTA survivors in orthopedic settings of Bahir Dar, Ethiopia.

S. No	Questions	Responses				
		Very poor	Poor	Fair	Good	Very good
1	In the past 7 days, my sleep quality was….	v38 (6.7)	61 (10.7)	101 (17.7)	213 (37.4)	157 (27.5)
In the past 7 days						
		Not at all	A little bit	Somewhat	Quite a bit	Very much
2	My sleep was refreshing.	35 (6.1)	76 (13.3)	191 (33.5)	165 (28.9)	103 (18.1)
3	I had a problem with my sleep.	56 (9.8)	98 (17.2)	226 (39.6)	102 (17.9)	88 (15.4)
4	I had difficulty falling asleep.	65 (11.4)	103 (18.1)	177 (31.1)	139 (24.4)	86 (15.1)
5	My sleep was restless.	85 (14.9)	102 (17.9)	170 (29.8)	109 (19.1)	104 (18.2)
6	I tried hard to get to sleep.	87 (15.3)	112 (19.6)	133 (23.3)	160 (28.1)	78 (13.7)
7	I worried about not being able to fall.	90 (15.8)	122 (21.4)	149 (26.1)	128 (22.5)	81 (14.2)
8	I was satisfied with my sleep.	28 (4.9)	56 (9.8)	143 (25.1)	178 (31.2)	165 (28.9)

### Factors Associated With PTSD

In bivariate analysis, sum score of Alcohol Use Disorder Identification Test (AUDIT), severe impact on social relationship and family functioning, severity of sleep problems, accident on other family members, witnessing death during the accident, and previous reported psychiatric illness was significantly associated with the incidence of PTSD. In the final model (multivariable analysis) witnessing death, severe sleep problem and severe impairment in family functioning were significantly associated with PTSD (**Table 6**).

**Table 6:** Factors associated with PTSD after RTA.

Variables	Responses	PTSD		RR	95% CI		P-value
		**No**	**Yes**		**Lower CI**	**Upper CI**	
Impact on family relationship	Mild	11	41	1.0			
	Moderate	23	16	1.12	0.70	1.78	0.64
	Markedly	27	35	1.41	.99	1.99	0.58
	**Extremely**	**99**	**47**	**2.18**	**1.62**	**2.92**	**<0.001**
Sleep problem	**None to slight**	**107**	**35**	**0.42**	**0.29**	**0.63**	**<0.001**
	Mild	41	83	0.99	0.73	1.33	0.92
	Moderate	12	21	1.0			
Death during accident	No	113	58	1.0			
	**Yes**	**47**	**81**	**1.31**	**1.03**	**1.68**	**0.03**
Bolded texts shows statistically significant results.							

## Discussion

The incidence of PTSD among RTA survivors was 46.5%. PTSD was associated with witnessing death during the accident, severe impact of the trauma on family relationship, and previous psychiatric illness.

The incidence of PTSD was higher in the current study than studies conducted elsewhere (9–12). The possible reason for this discrepancy may be:

1) In the current study, we include admitted survivors only. Individuals with severe injuries are more likely to be admitted, and PTSD was predicted by the severity of injuries in other studies (7, 8).
2) We exclude injuries who lasted for more than a month. Since, the level of stress decreases over time the high incidence may be explained by short duration (32). However, the level of PTSD is comparable with other studies in United Kingdom, Spain, and Greece (33, 34).

The most consistent associated factors with PTSD in previous studies include: gender, age, severity of injuries, premorbid psychological health, and perceived life threat (11, 32, 35, 36). In the current study, PTSD was associated with previous psychiatric illness, which was also reported in the study from UK (32, 33, 37).

There is 31% increased risk of developing PTSD in peoples who witnessed death than who don’t. This is also reported in other studies (2, 38). Impairment in family relationship and PTSD were significantly associated with PTSD (RR = 2.18, 95% CI [1.62, 2.92]). Severe impairment in family relationship results in serious psychological burden which results in worsening of PTSD symptoms (39, 40). Maintaining family relation especially in traditional society is important. If an individual fails to maintain this responsibility, it will worsen the psychological trauma (41, 42).

Some important variables such as sex and severity of injury were not associated with the incidence of PTSD. The result was mixed in previous evidences where some report higher level of PTSD symptoms among females and people with severe injuries while others report no significant association (14–16).

## Limitation

The study had some limitations. The first limitation is related with the validity of instruments, but we have done pretest and factor analysis to familiarize the tool, and the final version of the tools had excellent psychometric properties. The second limitation which may affect the result was we didn’t consider the injury type in the analysis which may limit application. We also excluded people with major cognitive impairment, and the result may be underestimated because these people have higher chance of having PTSD symptoms. We may also miss important biological predictors.

## Conclusion

Nearly half of RTA survivors develop PTSD after a month, but the treatment is merely focused on physical rehabilitation. This indicates that clinicians need to link these patients to the psychiatry clinic as it is also helpful for better physical rehabilitation. Special attention should be given to patients who witnessed death, with serious disability, and have previous psychiatric history.

## Data Availability

The original data will be made available by placing a reasonable request with the corresponding author.

## Ethics Statement

Ethical clearance was obtained from Bahir Dar University. The code was given to every participant and their address was kept private. Participants were informed about the aim of the study, the advantages of the study, confidentiality, and their rights even to stop in the middle of the interview. Written consent was taken before data collection. Individuals with PTSD were linked to the psychiatric clinic.

## Author Contributions

WF and TM conceived the research. They framed the methods, did the analysis, and wrote the final paper. HB participated in framing the method, data collection, and write-up. KY and AB conceived the research idea and approved the final manuscript. All the authors read and agreed on the final manuscript.

## Funding

The research was funded by Bahir Dar University. WF was supported through the DELTAS Africa Initiative [DEL-15-01]. The DELTAS Africa Initiative is an independent funding scheme of the African Academy of Sciences (AAS)’s Alliance for Accelerating Excellence in Science in Africa (AESA) and supported by the New Partnership for Africa’s Development Planning and Coordinating Agency (NEPAD Agency) with funding from the Wellcome Trust [DEL-15-01] and the UK government. The views expressed in this publication are those of the author(s) and not necessarily those of AAS, NEPAD Agency, WellcomeTrust, or the UK government.

## Conflict of Interest Statement

The authors declare that the research was conducted in the absence of any commercial or financial relationships that could be construed as a potential conflict of interest.
