# Supplementary material for: Incidence of Post-Traumatic Stress Disorder After Road Traffic Accident
Source: Front Psychiatry. 2019 Jul 19;10:519. doi: 10.3389/fpsyt.2019.00519 (PMC6659351; doi:10.3389/fpsyt.2019.00519)
Supplement: Supplementary file 1 [file DataSheet_1.pdf]

Correlation Matrix[a]

		P1Flashback	P2Nightmare	P3Daydream	P4IrritableforanymemoryoftheRTA	P5physiologicalchangeformemoryof	P6Trynottorememberthesituation	P7Avoidance	P8Amenstic	P9Lossofpleasure	P10Isolation	P11slowness	P12feelingoflosingthefuture	P13sleepproblem	P14Irritablity	P15Lossofconcentration	P16Hypervigilliant	P17Nervousness
Correlation	P1Flashback	1.000	.822	.762	.736	.730	.682	.658	.676	.605	.675	.651	.551	.493	.635	.657	.396	.652
	P2Nightmare	.822	1.000	.742	.714	.711	.685	.687	.676	.579	.685	.651	.525	.441	.593	.641	.374	.640
	P3Daydream	.762	.742	1.000	.720	.792	.724	.748	.736	.629	.695	.661	.579	.506	.638	.682	.374	.708
	P4IrritableforanymemoryoftheRTA	.736	.714	.720	1.000	.763	.700	.737	.696	.658	.732	.661	.551	.522	.634	.652	.326	.699
	P5physiologicalchangeformemoryof	.730	.711	.792	.763	1.000	.728	.768	.771	.643	.723	.728	.585	.570	.615	.723	.398	.771
	P6Trynottorememberthesituation	.682	.685	.724	.700	.728	1.000	.699	.670	.589	.645	.625	.529	.477	.544	.643	.365	.671
	P7Avoidance	.658	.687	.748	.737	.768	.699	1.000	.661	.625	.671	.652	.521	.485	.589	.636	.337	.669

	P8Amenstic	.676	.676	.736	.696	.771	.670	.661	1.000	.629	.732	.711	.576	.565	.622	.694	.377	.761
	P9Lossofpleasure	.605	.579	.629	.658	.643	.589	.625	.629	1.000	.613	.609	.561	.527	.547	.588	.424	.641
	P10Isolation	.675	.685	.695	.732	.723	.645	.671	.732	.613	1.000	.721	.554	.504	.636	.657	.432	.688
	P11slowness	.651	.651	.661	.661	.728	.625	.652	.711	.609	.721	1.000	.607	.578	.641	.675	.379	.701
	P12feelingoflosingthefuture	.551	.525	.579	.551	.585	.529	.521	.576	.561	.554	.607	1.000	.499	.538	.571	.432	.610
	P13sleepproblem	.493	.441	.506	.522	.570	.477	.485	.565	.527	.504	.578	.499	1.000	.526	.552	.483	.566
	P14Irritablity	.635	.593	.638	.634	.615	.544	.589	.622	.547	.636	.641	.538	.526	1.000	.609	.341	.628
	P15Lossofconcentration	.657	.641	.682	.652	.723	.643	.636	.694	.588	.657	.675	.571	.552	.609	1.000	.368	.738
	P16Hypervigilliant	.396	.374	.374	.326	.398	.365	.337	.377	.424	.432	.379	.432	.483	.341	.368	1.000	.327
	P17Nervousness	.652	.640	.708	.699	.771	.671	.669	.761	.641	.688	.701	.610	.566	.628	.738	.327	1.000
Sig. (1-tailed)	P1Flashback		.000	.000	.000	.000	.000	.000	.000	.000	.000	.000	.000	.000	.000	.000	.000	.000
	P2Nightmare	.000		.000	.000	.000	.000	.000	.000	.000	.000	.000	.000	.000	.000	.000	.000	.000
	P3Daydream	.000	.000		.000	.000	.000	.000	.000	.000	.000	.000	.000	.000	.000	.000	.000	.000

P4Irritableforanymemoryofthe RTA	.000	.000	.000		.000	.000	.000	.000	.000	.000	.000	.000	.000	.000	.000	.000	.000
P5physiologicalchangeformemoryof	.000	.000	.000	.000		.000	.000	.000	.000	.000	.000	.000	.000	.000	.000	.000	.000
P6Trynottorememberthesituation	.000	.000	.000	.000	.000		.000	.000	.000	.000	.000	.000	.000	.000	.000	.000	.000
P7Avoidance	.000	.000	.000	.000	.000	.000		.000	.000	.000	.000	.000	.000	.000	.000	.000	.000
P8Amenstic	.000	.000	.000	.000	.000	.000	.000		.000	.000	.000	.000	.000	.000	.000	.000	.000
P9Lossofpleasure	.000	.000	.000	.000	.000	.000	.000	.000		.000	.000	.000	.000	.000	.000	.000	.000
P10Isolation	.000	.000	.000	.000	.000	.000	.000	.000	.000		.000	.000	.000	.000	.000	.000	.000
P11slowness	.000	.000	.000	.000	.000	.000	.000	.000	.000	.000		.000	.000	.000	.000	.000	.000
P12feelingoflosingthefuture	.000	.000	.000	.000	.000	.000	.000	.000	.000	.000	.000		.000	.000	.000	.000	.000
P13sleepproblem	.000	.000	.000	.000	.000	.000	.000	.000	.000	.000	.000	.000		.000	.000	.000	.000
P14Irritablity	.000	.000	.000	.000	.000	.000	.000	.000	.000	.000	.000	.000	.000		.000	.000	.000

	P15Lossofconcentration	.000	.000	.000	.000	.000	.000	.000	.000	.000	.000	.000	.000	.000	.000		.000	.000
	P16Hypervigilliant	.000	.000	.000	.000	.000	.000	.000	.000	.000	.000	.000	.000	.000	.000	.000		.000
	P17Nervousness	.000	.000	.000	.000	.000	.000	.000	.000	.000	.000	.000	.000	.000	.000	.000	.000	

a. Determinant = 2.83E-007
